# Tracking airborne CO_2_ mitigation and low cost transformation into valuable carbon nanotubes

**DOI:** 10.1038/srep27760

**Published:** 2016-06-09

**Authors:** Jiawen Ren, Stuart Licht

**Affiliations:** 1 Department of Chemistry, George Washington University, Washington, DC, 20052, USA

## Abstract

Primary evidence of the direct uptake of atmospheric CO_2_ and direct transformation into carbon nanotubes, CNTs, is demonstrated through isotopic labeling, and provides a new high yield route to mitigate this greenhouse gas. CO_2_ is converted directly to CNTs and does not require pre-concentration of the airbone CO_2_. This C2CNT (CO_2_ to carbon nanotube) synthesis transforms CO_2-gas_ dissolved in a 750 °C molten Li_2_CO_3_, by electrolysis, into O_2-gas_ at a nickel electrode, and at a steel cathode into CNTs or carbon or nanofibers, CNFs. CNTs are synthesized at a 100-fold price reduction compared to conventional chemical vapour deposition, CVD, synthesis. The low cost conversion to a stable, value-added commodity incentivizes CO_2_ removal to mitigate climate change. The synthesis allows morphology control at the liquid/solid interface that is not available through conventional CVD synthesis at the gas/solid interface. Natural abundance ^12^CO_2_ forms hollow CNTs, while equivalent synthetic conditions with heavier ^13^CO_2_ favours closed core CNFs, as characterized by Raman, SEM and TEM. Production ease is demonstrated by the first synthesis of a pure ^13^C multiwalled carbon nanofiber.

One climate change mitigation pathway is to transform CO_2_ into a stable, valuable commodity, which will provide an incentive to consume smokestack or atmospheric CO_2_. Recently, we reported on a high yield synthesis of CNF carbon, nanofibers, from carbon dioxide in molten lithium carbonate^1^. This electrolytic synthesis provides a facile route for the transformation of the greenhouse gas into a high-value commodity^2^, and occurs at low energy with low electrolysis potentials^3^. Here, we show that the synthesis produces an even more valuable product, CNTs, carbon nanotubes, rather than CNFs, carbon nanofibers, and provide cost analysis, and critical evidence that the product is not synthesized by consuming the electrolyte, but rather that the CNT is formed directly from atmospheric CO_2_. As illustrated in Fig. 1, the synthesis transforms CO_2_ gas, dissolved in a molten carbonate electrolyte by electrolysis at a nickel anode and at a galvanized steel cathode. At the anode the product is O_2_ and at the cathode the product is uniform carbon nanofibers, CNFs, or CNTs, which are CNFs with hollow interiors. Due to their superior strength than steel, conductivity, flexibility and durability CNTs and CNFs have applications ranging from capacitors, Li-ion batteries, and nanoelectronics to the principal component of lightweight, high strength building materials, such as used in replacing steel and concrete in bridge construction, wind turbines, and lighter-weight structural materials for jets, cars, and athletic equipment.

In the C2CNT (CO_2_ to carbon nanotube) synthesis, metallic zinc at the galvanized steel initiates chemical carbon formation, and carbon nanofiber electrochemical growth is promoted by transition metal nucleation sites (such as nickel, iron, cobalt or copper). The nano-morphology is tuned by controlling the electrolysis conditions, such as the concentration of added alkali oxide, the temperature, the current density, and the transition metal concentration. Control of the latter variable is demonstrated both by the direct addition of transition metal oxides to the molten carbonate electrolyte, or (in the case of nickel) by the controlled low concentration release of nickel from the anode^1^. In the synthesis, the electrodes are conventional metals, and the lithium electrolyte if not consumed is inexpensive.

Critical to this alternative CTCNT pathway to transform the principal greenhouse gas into a stable, useful and valuable commodity is the demonstration that the product is not synthesized by consuming the carbonate electrolyte, but rather the CNF is formed directly from atmospheric CO_2_. Here, we use carbon isotope tracking to substantiate that atmospheric CO_2_ is directly incorporated into the synthesized carbon nanostructure. We also report on electrolysis conditions to yield the first synthesis of pure carbon 13 isotope multi-walled carbon nanofiber, MWCNF, and conditions which favor formation of either the carbon nanotube or carbon nanofiber morphology.

Whereas syntheses of single^4^,^5^,^6^,^7^,^8^,^9^ (SWCNT) and double^10^,^11^,^12^ (DWCNT) walled ^13^C isotope carbon nanotubes, quantum dots^13^ and two dimensional ^13^C nanomorphologies such as graphenes have been reported^14^,^15^,^16^,^17^, studies of ^13^C multiwalled carbon nanofibers have not been evident, presumably due to the complexity and expense of conventional electrospinning/carbonization or chemical vapor deposition syntheses which would require additional synthetic steps to form the requisite ^13^C polymers or ^13^C organometallic reactants. Similarly, density functional and molecular dynamic simulation studies are found on ^13^C isotope SWCNTs^18^,^19^, but not on nano-morphologies which would require modeling of a larger numbers of carbon centers. In general ^13^C isotopes have been of interest from the perspective of kinetic effects on catalysis, thermal conductivity, as well as on diffusion and NMR effects^18^,^20^,^21^.

Here, (i) through isotope tracing (^12^CO_2_ air into molten Li_2_^13^CO_3_ electrolyte) we demonstrate the direct uptake of gas phase CO_2_ and its transformation into carbon nanotubes at high yield. Atmospheric concentrations (0.04%) of CO_2_ are directly consumed and transformed, and this occurs without the pre-concentration that is required from CO_2_ sequestration processes. We present (ii) a short derivation of costs, and (iii) the first synthesis of a pure carbon 13 isotope multiwalled carbon nanofiber, MWCNF. (iv) Finally, we show that under the electrolytic synthesis conditions studied, natural abundance ^12^CO_2_ favours formation of CNTs, while equivalent synthetic conditions with heavier ^13^CO_2_ favours formation of (solid core) carbon nanofibers.

## Experimental

Experimental details of the solar thermal electrochemical process, STEP, synthesis of a variety of societal staples and carbon capture have been delineated in previous publications^22^,^23^,^24^,^25^,^26^,^27^,^28^,^29^,^30^. This study focuses primarily on the STEP for carbon electrochemical reactor component to form a high-yield CNF component.^13^C lithium carbonate (Sigma-Aldrich, 99 atom% ^13^C) ^13^C carbon dioxide (Sigma-Aldrich, 99 atom% ^13^C), lithium carbonate (Alfa Aesar, 99%) and lithium oxide (Alfa Aesar, 99.5%) are combined to form various molten electrolytes.

Electrolyses are driven galvanostatically, at a set constant current as described in the text. The electrolysis is contained in a pure nickel 100 ml crucible (Alfa Aesar). Electrolyses in the Ni crucible used the inner walls of the crucible as the anode. A wide variety of steel wires for coiled cathodes are effective, an economic form (used in this study) is Fi-Shock 14 gauge, galvanized steel wire model #BWC-14200. Following an initial low current (0.05 A for 10minutes, 0.1A for 10 minutes, 0.2A for 5 minutes, and 0.4A for 5 minutes) step to grow Ni nucleation sites on the cathode, CNTs or CNFs are grown on an immersed 10 cm^2^ galvanized steel cathode at 1.00 A for 1 hour. The electrolysis potentials are consistent with those we have recently reported for generic carbon deposition in a molten Li_3_CO_3_ electrolyte as a function of current density, and as previously reported, the potential decreases with addition of Li_2_O to the electrolyte^3^. Two nanostructures are generated, straight CNTs or CNFs that are grown in electrolyte without added Li_2_O, or tangled CNTs or CNFs that are grown when Li_2_O has been added to the electrolyte. During electrolysis, the carbon product accumulates at the cathode, which is subsequently removed and cooled. Subsequent to electrolysis the product remains on the cathode, but falls off with electrolyte when the cathode is extracted, cooled, and uncoiled. The product is washed with 11 m HCl, and separated from the washing solution by centrifugation.

The carbon product is washed, and analyzed by PHENOM Pro-X Energy Dispersive Spectroscopy (EDS) on the PHENOM Pro-X SEM or Carl Zeiss Sigma VP Field Emission SEM and by TEM with a JEM 2100 LaB6 TEM. Raman spectroscopy was measured with a LabRAM HR800 Raman microscope (HORIBA) with 532.14 wavelength incident laser light, with a high resolution of 0.6 cm^−1^.

### C2CNT cost analysis

CO_2_ removal here occurs by the electrolytic reduction of tetravalent carbon in proximity to the cathode to solid, zerovalent carbon. The concentration of tetravalent carbon available for reduction is much higher as carbonate than as airborne or soluble CO_2_. Molten carbonate, such as Li_2_CO_3_(liquid), contains ~10 molar reducible tetravalent carbon/liter. Air contains 0.04% CO_2_, equivalent to 1 × 10^−5 ^molar of tetravalent carbon/liter. Hence, molten carbonates formed by the dissolution of atmospheric CO_2_, and its conversion to carbonate, provide a spontaneous, million-fold concentration increase of reducible tetravalent carbon sites per unit volume, compared to air, facilitating the direct conversion of atmospheric CO_2_. In this process, the production of carbon, such as CNFs, or their hollow morphology, CNTs, by electrolysis in lithium carbonate occurs simultaneously with the production of oxygen and dissolved lithium oxide:


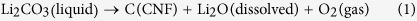


Li_2_CO_3_ consumed by electrolysis is continuously replenished by reaction of this excess Li_2_O, formed as a product in the Equation 1 electrolysis, with CO_2_ from the air (or CO_2_ available in higher concentration from stack emissions):


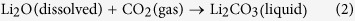


for the net reaction (combining Equations 1 and 2):


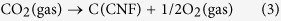


This C2CNT cost analysis assumes a generic renewable electric supply, and does not include the additional efficiency advantages of a solar thermal and electric supply^22^,^23^,^24^,^25^,^26^,^27^,^28^,^29^,^30^. Electrolysis costs to produce CNFs, or CNTs, will be similar to infrastructure costs associated with the chlor-alkali and aluminum industries. The electrical energy costs are low, requiring 0.9 to 1.4 V^3^. CNFs or CNTs are consistently prepared here at 80 to 100% coulombic efficiency of the four electrons required to reduce CO_2_, which at $0.10 per kWh is equivalent to $800 to $1,600 per metric tonne of CNT. After washing off the electrolyte, the product consists of >80% pure carbon nanotubes^1^. The synthesis continues to be refined for higher purities and specific morphologies. The electrolyte tends to tenaciously bind onto the CNT product, but is water soluble, and purification does not require toxins or costly washing materials. Analogous waste management and cleaning costs of recyclables (plastics and metals) is < < $10 per tonne, and is assumed to be similar here. Lithium carbonate is not consumed during the CO_2_ electrolysis and at today’s cost of $6,000 per tonne, as amortized over ten year’s usage, cost an additional $140 per metric tonne CNT, additionally the amortized cost of the nickel and steel electrodes and ancillary equipment is low for a combined cost upper limit of ~$2,000 per metric tonne of CNT. These costs compare to today’s conventional chemical vapor deposition or electrospun production cost of ~$25,000 per tonne of CNF^2^ and $200,000 to 400,000 per metric tonne of industrial grade (90% purity) CNTs^31^. The low costs of C2CNT production opens high revenue windows and provides a significant incentive for CO_2_ removal, and as costs decrease also provides impetus for CNF and CNT market growth.

### Raman isotope spectra and tracking the C2CNT transformation

**Figure 2:**
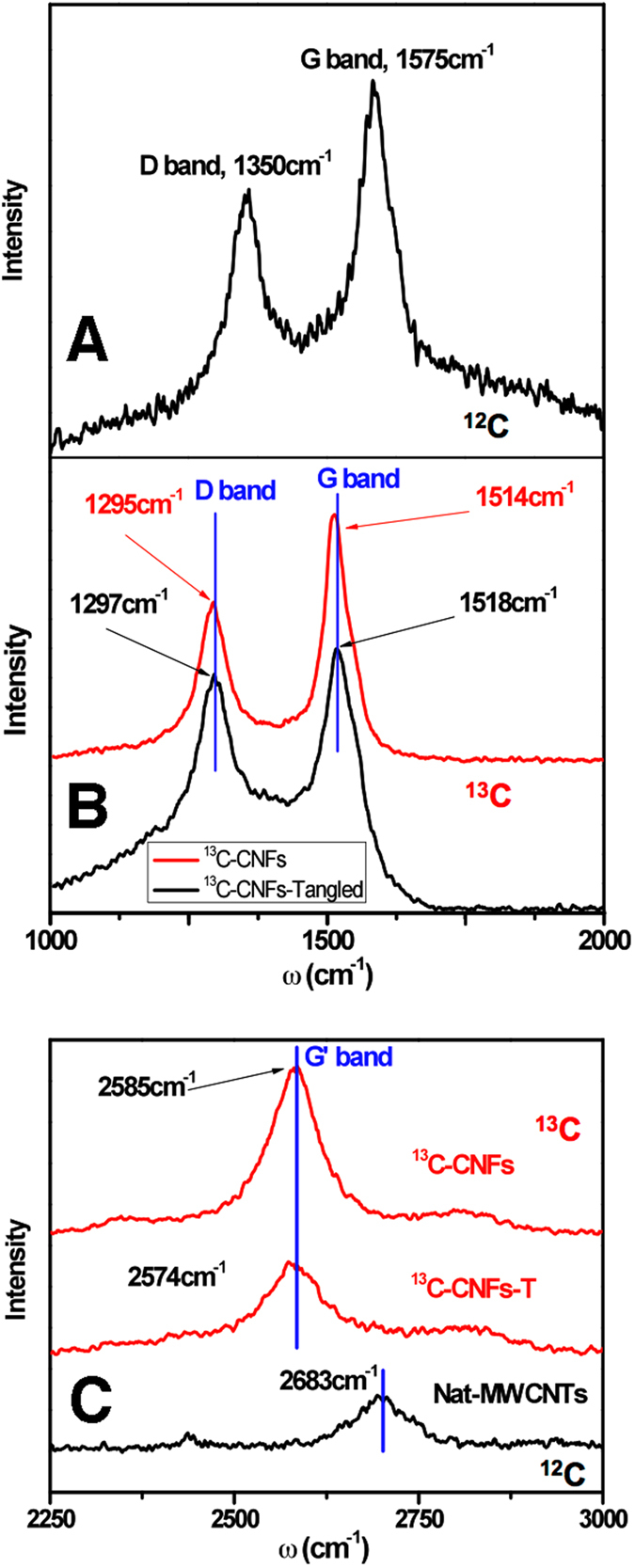
Raman spectroscopy comparison of natural abundance (99% ^12^C) and ^13^C carbon nanotubes/carbon nanofibers. As synthesized at 750 °C by the electrolysis of CO_2_ in Li_2_CO_3_ electrolytes at a 10 cm^2^ galvanized cathode with a Ni anode. The carbon isotope composition of the CO_2_ and the Li_2_CO_3_ is either ^13^C or natural abundance ^12^C as described in the text.

Figure 2 presents Raman spectra of carbon nanofibers that yield structural and isotope replacement information. Each was synthesized by electrolysis under identical conditions except one (top, Fig. 2A) was produced with natural abundance CO_2_ and Li_2_CO_3_, while others (middle, Fig. 2B) were grown in molten ^13^C Li_2_CO_3_. The natural abundance carbon nanotubes (Fig. 2A) Raman spectrum exhibits two sharp peaks at 1350 cm^−1^ and 1575 cm^−1^, which correspond to the disorder-induced mode (D band) and the high frequency E_2g_ first order mode (G band), respectively. The intensity ratio between D band and G band (I_D_/I_G_) is an important parameter to evaluate the graphitization, and here the ratio of 0.70, is consistent with that of commercial hollow carbon natural abundance carbon isotope nanofibers^32^.

The Raman spectrum observed for ^13^C-CNFs in Fig. 2B have both bands downshifted compared to the ^12^C spectrum in Fig. 2A. The frequency of Raman modes in samples containing a concentration, C, of ^13^C is determined by the following equation which incorporates the increased mass of this isotope:^11^


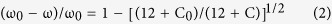


In Equation 2, ω_0_ is the frequency of a particular mode in a natural abundance CNT sample, ω is the frequency of a particular mode in ^13^C enriched carbon sample, and C_0_ = 0.0107 is the natural abundance of ^13^C. In accord with Equation 2, the D and G band for pure ^13^C-CNFs should peak at 1297 and 1514 cm^−1^, respectively. The close agreement between the theoretical calculated (1297 and 1514 cm^−1^) Raman peak position and the observed experimental peak (red curve, Fig. 2B) indicates the CNFs obtained approached 100% ^13^C isotope enrichment. The intensity ratio of I_D_/I_G_ is 0.63, indicating a better degree of graphitization than for the natural-enriched CNTs (Fig. 2A).

Generally in this study during the electrolyses, either ^13^CO_2_ was exposed to Li_2_^13^CO_3_, or (natural abundance) ^12^CO_2_ was exposed to Li_2_^12^CO_3_. Our calculations indicated that atmospheric CO_2_ can be absorbed and then converted into carbon with Li_2_O present in carbonate melts, by low energy electrolysis with the potential constrained by the reaction: Li_2_O + 2CO_2 _→ Li_2_CO_3_ + C + O_2_^3^.

The hypothesis that the CNFs are formed directly from absorbed atmospheric CO_2_ via Equation 3 is significant as it provides a direct process to mitigate this greenhouse gas. The hypothesis is tested here by a study in which a ^13^C electrolyte (1 m Li_2_O in Li_2_^13^CO_3_) is exposed to regular (natural abundance, 99% ^12^C) air containing 0.04% CO_2_ during the electrolysis. This experiment is conducted to determine if this carbon 12 from CO_2_ gas in the air was incorporated into the electrolyzed product. All other steps in the procedure were identical to the ^13^CO_2_ exposed synthesis. The product’s Raman spectrum is shown in the black curve in the Fig. 2B, and is similar, but slightly up-shifted compared to the red curve spectrum, that of pure ^13^C-CNFs. The G-band shift is ~4 cm^−1^ towards higher frequency, indicating ~4% ^12^C was present in this sample using Equation 1, to provide evidence that (^12^C) CO_2_ is directly absorbed from the air in the formation of the carbon product. A broadening of the G-band in this sample, which can be seen by the larger FWHM (full width at half maximum) is further evidence of a ^12^C/^13^C mixture, because in the other, pure ^12^C, or pure ^13^C, cases the G-band is single peak and the FWHM is the most narrow. This result acts to confirm our proposed mechanism that the presence of Li_2_O in the electrolyte absorbs the greenhouse gas CO_2_ from air and transforms it by electrolysis into CNFs.

The Raman G’ band of the products is presented in Fig. 2C. The peak frequency of pure ^13^C-CNFs is 2585 cm^−1^. The reported frequencies of the G’ band vary in the literature. For example, in ref. 4 the G’ band of SWCNTs is observed at 2526 cm^−1^, while in ref. 6 this band of SWCNTs at ~2580cm^−1^; both using 532 nm incident laser. The discrepancy may be due to a variation in morphology (such as zig-zag or arm-chair) and/or the relatively broad character of the peak. Our results are close to those in ref. 6.

### Isotope variation of nanostructure morphology: ^13^CNFs and ^12^CNTs

We had previously established that multiwalled carbon nanofibers are deposited at high yield when carbon dioxide, dissolved in lithiated molten carbonates is reduced at a galvanized steel electrode in the presence of nickel (or other transition metal) oxides^1^. Specifically, we had observed that a transition metal oxide that is highly soluble in the lithium carbonate electrolyte, such as iron oxide, results in an electrolysis product consisting of a wide array of nanofiber morphologies, while a limited solubility transition metal oxide, such as nickel oxide, results in a highly uniform product consisting of either straight (without added Li_2_O) or tangled (in an electrolyte with excess, dissolved Li_2_O) nanofibers. The extent to which the fibers had a hollow core (nanotubes) or a solid core (nanofibers) was not discussed in that study. Other physical chemical parameters are expected to influence the synthesized fiber nanomorphology. Under identical electrosynthetic conditions, the heavier mass of the molten ^13^C carbonate anion compared to the ^12^C (natural abundance) will affect the relative rate of mass diffusion of carbonate towards the cathode compared to the rate of oxide away from the cathode, during the carbon (IV) reduction and hence might be expected to influence the morphology of the product nanofibers. This expectation is tested in this study.

**Figure 3:**
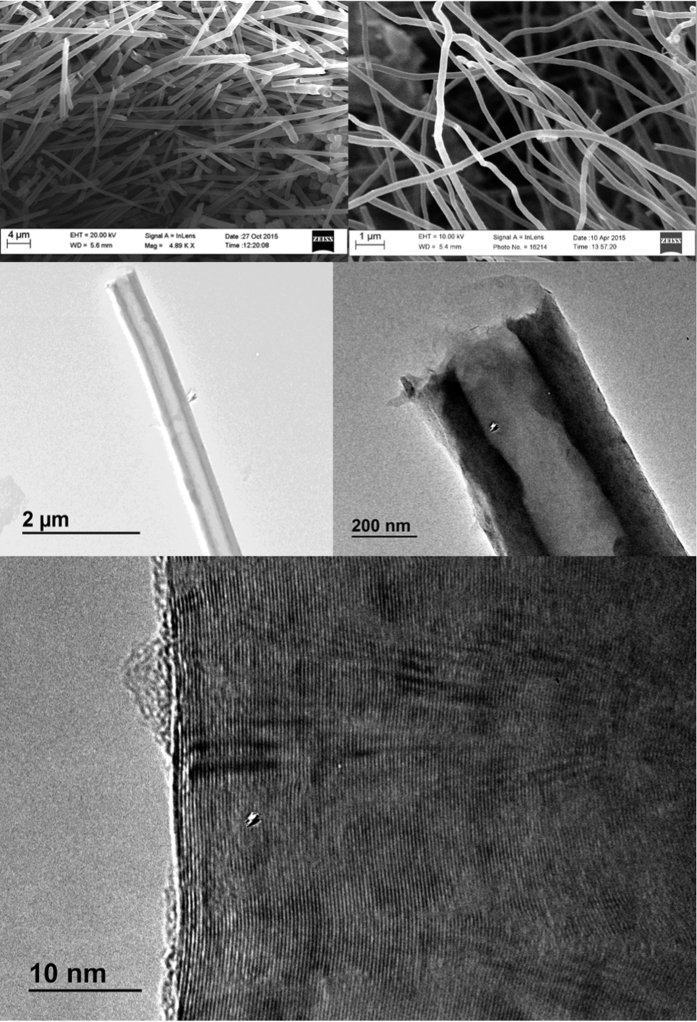
SEM and TEM microscopy of natural abundance (99% ^12^C) carbon nanotubes synthesized. by the electrolysis of carbon dioxide in molten lithium carbonate (at 750 °C without Li_2_O added to the Li_2_CO_3_ electrolyte on a 10 cm^2^ galvanized steel electrode with a Ni anode).

**Figure 4:**
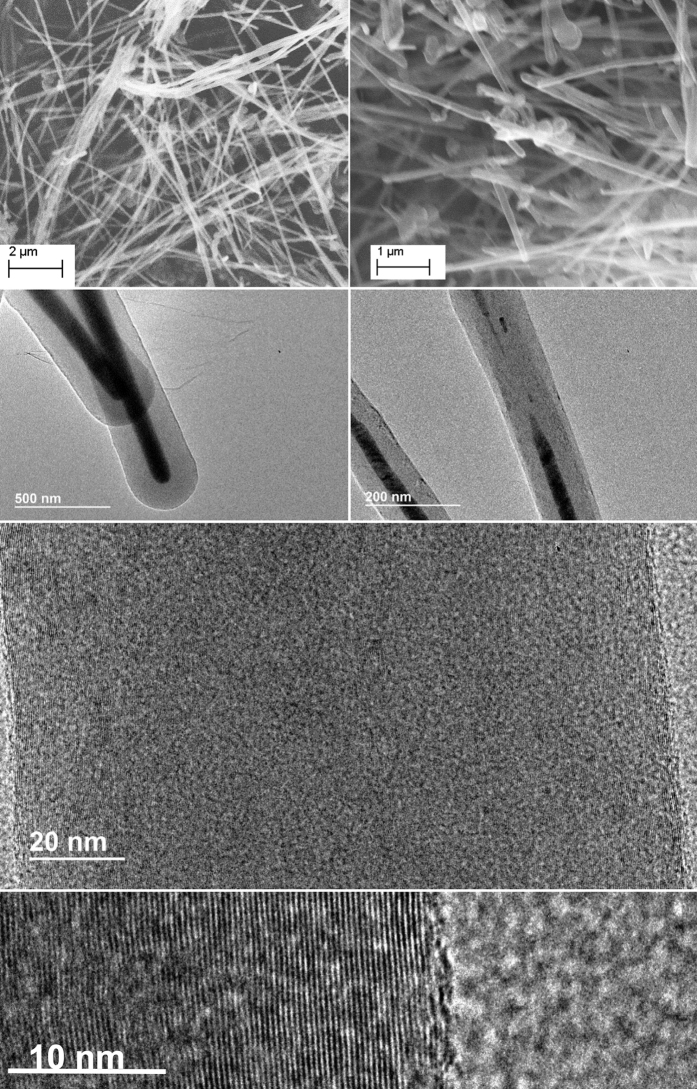
SEM and TEM microscopy of straight (99% ^13^C) carbon nanofibers. synthesized by the electrolysis of ^13^C carbon dioxide in 750 °C molten ^13^C lithium carbonate on a 10 cm^2^ galvanized steel electrode with a Ni anode.

Figures 3 and 4 present microscopy comparing the nanomorphology when ^12^C is the dominant isotope (Fig. 3) and when ^13^C is the dominant isotope (Fig. 4) used in the electrolytic synthesis. Here we observe that carbon fibers formed with the natural isotope CO_2_ consistently exhibit hollow cores (carbon nanotubes), whereas those formed from ^13^CO_2_ have solid or nearly solid cores (carbon nanofibers).

Figure 3 shows the typical SEM/TEM images of the multiwalled carbon nanotubes, MWCNTs, electrochemically synthesized from natural abundance CO_2_ in molten, natural abundance Li_2_CO_3_. Typically the MWCNTs have wall thickness of ~100–150 nm and inner diameter of ~160–210 nm. The distance between graphene layers from high resolution TEM is 0.342nm and therefore the wall contains about 300~450 layers. The layer spacing is consistent with pure ^12^C graphitic structures. We observe larger diameter nanotubes (with the same graphene layer spacing) when, rather than a galvanostatic DC current of 1 A (through the 10 cm^2^ galvanized steel cathode), the CNT growth current is pulsed at a low frequency (cycled at 9 minutes on (1 A), 1 minute off (0 A).

**Figure 5:**
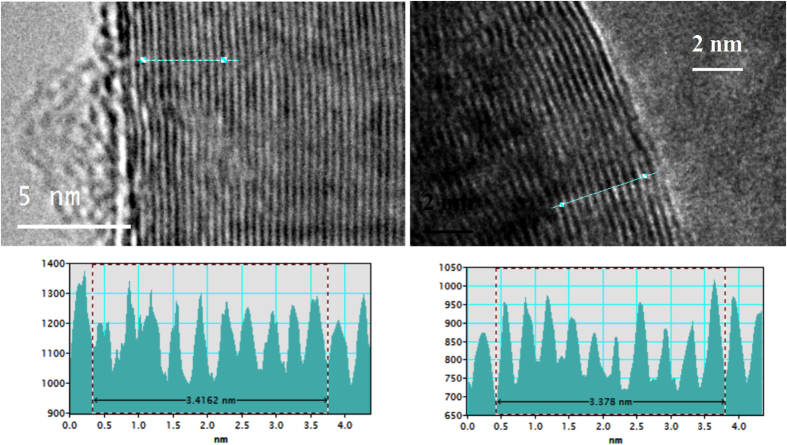
Representative examples of repeat TEM determination of interspatial MWCNTs graphene layer separation. in either ^13^C (left) or natural abundance carbon (right) grown by CO_2_ splitting in molten lithium carbonate.

As seen in Fig. 4, rather than the large core nanotubes produced by electrolysis from natural abundance (99% ^12^C) carbonate, the fibers grow with a nearly solid core structure from ^13^C (from ^13^CO_2_ in Li_2_^13^CO_3_), except where the nickel catalyst remains present, as analyzed by EDS. The separation between graphene layers in graphite is approximately 0.335 nm^33^, and can vary near this value in multiwalled carbon nanotubes. The spacing between carbon layers is seen to decrease from 0.342 nm for the natural abundance carbon nanostructures in Fig. 3, to 0.338 nm as shown in Fig. 4. Higher resolution TEM of the intergraphene layer spacing calculations of natural abundance carbon (left) compared to the ^13^C isotope (right) spacings shown in Fig. 5, and interspatial graphene spatial layer separations are determined by repeat measurements on TEM analyses. The ^13^C products exhibit a carbon nanofiber morphology with an inner (void) volume that is less than 6% of the total. Specifically, the ^13^C-CNFs have an outer diameter of ranging from 150 to 350 nm, a wall thickness of 75 to 175 nm. The observed yield of fibers from the ^13^C synthesis is 60–80% during replicate measurements, while from the natural abundance C synthesis, the observed carbon nanotube yield is typically 80–90%. The slightly lower yield for the ^13^C synthesis may be related to the higher cost of the reactants, which precluded further (than three) replicate measurements or optimizations, and as previously observed for natural carbon abundance electrolyses (as described in the supplementary information of ref. 1) the remaining product includes amorphous graphites and graphenes.

The thicker wall and smaller diameter of the ^13^C products compared to the ^12^C carbon nanotubes may be due to the different diffusion behaviours in Li_2_^13^CO_3_ electrolyte. CO_2_ is solvated in solution as carbonate, and diffusion of carbonate to the cathode supplies carbon for the nanotube/nanofiber growth. The heavier ^13^CO_3_^2−^ species in the ^13^C electrolyte has a lower mobility with respect to ^12^CO_3_^2−^ in the natural abundance carbonate electrolyte and this can influence (i) the distribution of the transition metal nucleation sites, (ii) the curvature of the carbon cap growing on the nucleation site, and (iii) the availability of carbon during the growth process. With the ^13^C nanostructures we observe by EDS, nickel both at the exterior and within the interior of the nanofiber (dark areas in the middle of Figs 4 and 5), and these inner core nucleation sites may promote additional inner core wall growth.

It is likely that the closer-spaced walls observed in the ^13^C compared to ^12^C synthesized structures are indicative of a more stable structure, which would be consistent with the observed tendency of the ^13^C to form more layers within the carbon nanotube matrix. Two physical chemical characteristics have been suggested that could be linked to our observation that multi-walled carbon nanotubes synthesized by the electrolysis of CO_2_ in molten carbonate are nearly solid (CNFs) when formed from carbon-13, but are hollow (CNTs) when formed from natural abundance carbon. These two characteristics are (i) the increased stability evident in the observed closer packed walls in the ^13^C CNFs and (ii) the lower mobility of the heavier ^13^CO_3_^2−^ compared to ^12^CO_3_^2−^. Whether either, or both of these characteristics dominate the growth mechanism which leads to these morphological difference requires further extended study. The 1.7% mass difference between ^13^CO_3_^2−^ compared to ^12^CO_3_^2−^ increases the relative rate at the growing carbon nanostructure on the cathode that electrogenerated oxide exits compared to the rate at which carbonate arrives and is consumed. Density functional calculations will be of interest to probe the hypothesis that heavier carbon isotopes would tend to form a smaller diameter carbon cap for carbon nanofiber growth, future molecular dynamic simulation studies will be of interest to probe the hypothesis that slower carbonate mobility will promote a more densely packed multiwalled carbon growth.

### Tangled compared to straight ^13^CNF morphology

Raman spectroscopic analysis was conducted to determine the degree of graphitization of the synthesized carbon nanostructures. In Fig. 2B, the D band peak correspond to the disorder-induced vibration and the higher frequency E_2g_ first order vibration mode G band peak corresponds to the graphitization vibration, and generally to sp^3^ and sp^2^ hybridized carbon species, respectively. The intensity ratio between D band and G band (I_D_/I_G_), is an important parameter to evaluate the graphitization and hence the total relative ratio of defective carbons in the material. The addition of Li_2_O to the synthesis electrolyte leads to the observed curvature of the conventional isotope ^12^C carbon nanotubes, and it is of interest there to investigate whether a similar occurrence here with the ^13^C carbon nanofibers, hence ^13^C carbon nanofibers were additionally grown with the addition of 1 m Li_2_O to the 750 °C Li_2_CO_3_ electrolyte. As shown in Fig. 2B, the I_D_/I_G_ ratio of 0.9 for tangled ^13^CNFs is significantly higher than the 0.6 ratio for the straight ^13^CNFs

At 750 °C, “pure” lithium carbonate spontaneously decomposes to form an equilibrium concentration of 0.3 m Li_2_O in Li_2_CO_3_^3^. This occurs through the reverse of Equation 2 that is the dissociation of the molten carbonate to form an equilibrium concentration: Li_2_CO_3_ ⇌ Li_2_O + CO_2_^3^. Experimentally, we estimate K_Li2CO3_ by mass loss or mass gain of the molten carbonate. Li_2_CO_3_ heated to 750 °C in air (containing p_CO2_ = 4.0 × 10^−4 ^atm CO_2_) evolves 0.017 mole fraction of CO_2_. The mass loss is equivalent to the equilibrium formation of 0.25 m Li_2_O (m ≡ molal = mol kg^−1^ Li_2_CO_3_) determined as 0.018/((1–0.018)*0.07391) kg mol^−1^ Li_2_CO_3_) in the molten lithium carbonate. However, this is regarded as a lower bound of this value to the equilibrium concentration at 750 °C value, as it does not include any initial Li_2_O impurities in the Li_2_CO_3_. pH titration by HCl of the original Li_2_CO_3_ dissolved as an aqueous solution indicates any Li_2_O impurity is <1% of the total carbonate. In accord with the reverse reaction of eq 5, when Li_2_O is added to the carbonate, the 750 °C Li_2_CO_3_ is observed to gain, rather than lose mass, that is absorb CO_2_ from air. We measure that a mix of 0.51 m Li_2_O in Li_2_CO_3_ heated to 750 °C gains mass equivalent to the absorption of 0.18 m CO_2_, for a final concentration of 0.33 m Li_2_O in solution. From the upper and lower bound measurements, the equilibrium concentration of Li_2_O in molten lithium carbonate at 750 °C is 0.29 (±0.04) m. This yields an experimental value of the 750 °C equilibrium constant of K_Li2CO3_ = 4.0 × 10^−4^ * 0.29 = 1.2 × 10^−4^.

**Figure 6:**
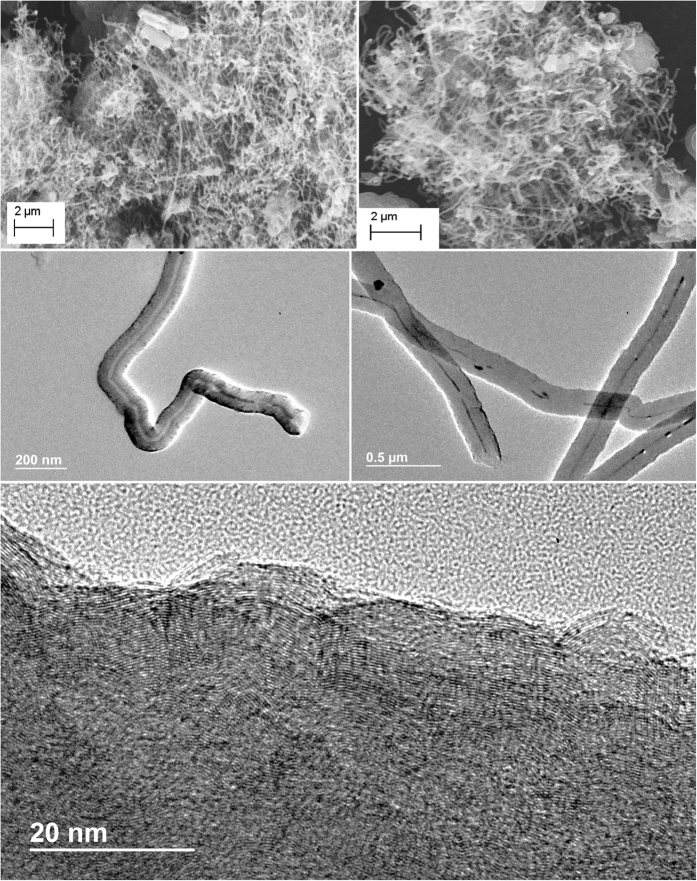
SEM and TEM microscopy of ^13^C tangled carbon nanofibers. synthesized in a high oxide environment by the electrolysis of ^13^C carbon dioxide in 750 °C molten ^13^C lithium carbonate containing 1 m Li2O on a 10 cm^2^ galvanized steel electrode with a Ni anode.

Higher concentrations of Li_2_O can be added to the electrolyte prior to the electrolysis and have two effects: (i) increased CO_2_ absorption from the gas and (ii) (as we had observed previously for natural abundance carbon) the high oxide environment near the cathode led to tangled, rather than straight, nanofiber growth. Here, this high oxide effect also occurs for the ^13^C carbon nanostructures. SEM/TEM were also conducted to study the structure properties of carbon nanofibers grown such a high oxide (1 m Li_2_O) environment, and are shown in Fig. 6. Instead of straight CNFs or MWCNTs, curved or tangled CNFs were obtained. Again, the inner void is very small and sometimes some nickel was incorporated into these inner voids.

The I_D_/I_G_ is 0.9 in the Raman spectrum for the 13 CNFs grown in the 1 m Li_2_O 750 °C Li_2_CO_3_ electrolyte, indicate a lesser degree of graphitization and many more defects. This is confirmed by HRTEM in the lower portion of Fig. 6, that the tangled ^13^C nanomorphology, as synthesized from the high oxide electrolyte, exhibits a less ordered, curved, pitted structure, in other words, containing many more defects than the (straight) ^13^C-MWCNFs and ^12^C-MWCNTs. As we will show in an upcoming study, synthetic control of the D:G ratio in these nanostructured carbon material is useful to engineer and improve the intercalation properties for Li-anode and Na-anode of carbon-based electrodes.

## Conclusions

We present a new high yield pathway to produce fully carbon 13 isotope enriched multiwalled carbon nanofibers in a straightforward synthesis directly from ^13^CO_2_ dissolved in Li_2_^13^CO_3_ melt at inexpensive galvanized steel and nickel electrodes. The heavier mass of ^13^C-carbonate ions results in different diffusion conditions in the bulk electrolyte and during the electrolytic growth at the liquid/solid interfaces, resulting in a high yield ^13^C multiwalled carbon nanofiber morphology, rather than a high yield multiwalled carbon nanotube morphology observed when instead synthesized from natural abundance (99% ^12^C) carbon. Raman spectra, SEM and TEM indicate the successful synthesis of ^13^C-CNFs and also provide insight into the role of CO_2_ as a reactant during the nanocarbon growth from reduction of carbonates by electrolysis. The direct transformation of gas phase CO_2_ to carbon nanofibers is demonstrated through the isotopic tracking of (natural abundance) carbon ^12^CO_2_ introduced into a carbon-13 (L_2_^13^CO_3_ molten electrolyte) media. Electrolyte dissolved Li_2_O serves as the absorbent for gas phase CO_2_ to add to the carbonate in the electrolyte. Li_2_O is released to the electrolyte during CNF formation to absorb further CO_2_ for the electrolytic formation of the MW^13^CNF.

Despite their superior mechanical, thermal and electronic properties, carbon nanotubes applications had been limited to date by the high cost of their conventional chemical vapour deposition synthesis. This CNT cost is higher than for carbon fibers, and 2 to 3 orders of magnitude higher than that of conventional graphitic or amorphous carbons^31^,^34^,^35^. Currently, bulk purified MWCNTs are sold for less than $200,000 per metric tonne, which is 10-fold greater than the cost of commercially available carbon fiber^36^. Hence, the finding here that the expensive MWCNT (hollow core) morphology product tends to be formed from the inexpensive ^12^CO_2_ reactant, while the less expensive product MWCNF (solid core) morphology tends to be formed from the expensive ^13^CO_2_ reactant provides an additional economic incentive to remove CO_2_ from the air or from stack emissions. Here, the facile synthesis of CNTs from natural abundance CO_2_, at high yield by electrolysis in molten carbonate at cost effective nickel and steel electrodes, provides an incentive to transform the greenhouse gas carbon dioxide into a useful, stable, valuable commodity.

## Additional Information

**How to cite this article**: Ren, J. and Licht, S. Tracking airborne CO_2_ mitigation and low cost transformation into valuable carbon nanotubes. *Sci. Rep.* **6**, 27760; doi: 10.1038/srep27760 (2016).

## Figures and Tables

**Figure 1:**
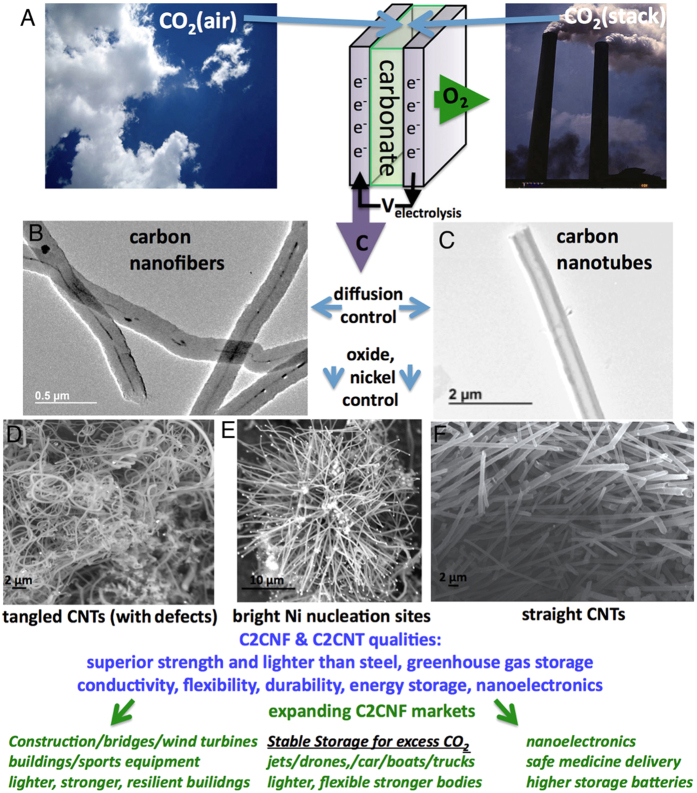
(**A**) High yield electrolytic synthesis of carbon nanostructures from dissolved air or smoke stack concentrations of CO_2_ in molten lithiated carbonates. During CO_2_ electrolysis, transition metal deposition controls the nucleation and morphology the carbon nanostructure. (**B,C**) Diffusion controls the formation of either nanotube (**C**: as grown from natural abundance CO_2_) or nanofiber (**B**: from ^13^C) morphologies. (**D–F**) Electrolytic oxide controls the formation of tangled (**D**: high Li_2_O) or straight (**F**: no added Li_2_O) nanotubes. (**E**) Bright spots are identified as Ni (by EDS) nucleation sites of carbon nanostructure growth. The carbon nanofiber and nanotubes provide high conductivity and superior carbon composite lightweight structural materials for jets, bridges, wind turbines, and electric vehicle bodies and batteries.
